# Dietary Compound Isoliquiritigenin, an Antioxidant from Licorice, Suppresses Triple-Negative Breast Tumor Growth via Apoptotic Death Program Activation in Cell and Xenograft Animal Models

**DOI:** 10.3390/antiox9030228

**Published:** 2020-03-10

**Authors:** Po-Han Lin, Yi-Fen Chiang, Tzong-Ming Shieh, Hsin-Yuan Chen, Chun-Kuang Shih, Tong-Hong Wang, Kai-Lee Wang, Tsui-Chin Huang, Yong-Han Hong, Sing-Chung Li, Shih-Min Hsia

**Affiliations:** 1 School of Nutrition and Health Sciences, College of Nutrition, Taipei Medical University, Taipei 11031, Taiwan; phlin@tmu.edu.tw (P.-H.L.); yvonne840828@gmail.com (Y.-F.C.); hsin246@gmail.com (H.-Y.C.); ckshih@tmu.edu.tw (C.-K.S.); sinchung@tmu.edu.tw (S.-C.L.); 2 School of Dentistry, College of Dentistry, China Medical University, Taichung 40402, Taiwan; tmshieh@mail.cmu.edu.tw; 3 Department of Dental Hygiene, College of Health Care, China Medical University, Taichung 40402, Taiwan; 4 Tissue Bank, Chang Gung Memorial Hospital, Tao-Yuan 33305, Taiwan; cellww@adm.cgmh.org.tw; 5 Graduate Institute of Health Industry Technology, Chang Gung University of Science and Technology, Tao-Yuan 33305, Taiwan; 6 Department of Nursing, Ching Kuo Institute of Management and Health, Keelung City 20301, Taiwan; kellywang@tmu.edu.tw; 7 Graduate Institute of Cancer Biology and Drug Discovery, College of Medical Science and Technology, Taipei Medical University, Taipei 11031, Taiwan; tsuichin@tmu.edu.tw; 8 Department of Nutrition, I-Shou University, Kaohsiung City 82445, Taiwan; yonghan@isu.edu.tw; 9 Graduate Institute of Metabolism and Obesity Sciences, College of Nutrition, Taipei Medical University, Taipei 11031, Taiwan; 10 School of Food Safety, College of Nutrition, Taipei Medical University, Taipei 11031, Taiwan; 11 Nutrition Research Center, Taipei Medical University Hospital, Taipei 11031, Taiwan

**Keywords:** isoliquiritigenin (ISL), triple-negative breast cancer, apoptosis, autophagy

## Abstract

Patients with triple-negative breast cancer have few therapeutic strategy options. In this study, we investigated the effect of isoliquiritigenin (ISL) on the proliferation of triple-negative breast cancer cells. We found that treatment with ISL inhibited triple-negative breast cancer cell line (MDA-MB-231) cell growth and increased cytotoxicity. ISL reduced cell cycle progression through the reduction of cyclin D1 protein expression and increased the sub-G1 phase population. The ISL-induced apoptotic cell population was observed by flow cytometry analysis. The expression of Bcl-2 protein was reduced by ISL treatment, whereas the Bax protein level increased; subsequently, the downstream signaling molecules caspase-3 and poly ADP-ribose polymerase (PARP) were activated. Moreover, ISL reduced the expression of total and phosphorylated mammalian target of rapamycin (mTOR), ULK1, and cathepsin B, whereas the expression of autophagic-associated proteins p62, Beclin1, and LC3 was increased. The decreased cathepsin B cause the p62 accumulation to induce caspase-8 mediated apoptosis. In vivo studies further showed that preventive treatment with ISL could inhibit breast cancer growth and induce apoptotic and autophagic-mediated apoptosis cell death. Taken together, ISL exerts an effect on the inhibition of triple-negative MDA-MB-231 breast cancer cell growth through autophagy-mediated apoptosis. Therefore, future studies of ISL as a supplement or alternative therapeutic agent for clinical trials against breast cancer are warranted.

## 1. Introduction

Breast cancer is the most frequently diagnosed form of cancer and the leading cause of cancer-related deaths in women worldwide [1,2]. As a result of advanced and multidisciplinary therapeutic treatments, the five-year survival rate of breast cancer has greatly improved [3]. However, an increasing trend in the incidence rate of breast cancer has been observed, and the age of patients who are diagnosed with breast cancer has been reported to be trending younger [4].

Breast cancer is categorized into three major subtypes based on the presence or absence of molecular markers for estrogen receptors (ERs), progesterone receptors (PRs), and human epidermal growth factor 2 (ERBB2, formerly HER2/neu). The standard therapy for all patients with breast cancer is tumor eradication by surgical resection. Then, a systemic therapeutic strategy for breast cancer is determined by tumor stage and subtype. Patients with hormone-receptor-positive tumors receive endocrine therapy, such as treatment with hormone receptor antagonists, in combination with limited chemotherapy. For ERBB2-positive tumors, patients can receive ERBB2-targeted antibodies or a combination of small-molecule inhibitors and chemotherapy. Patients with triple-negative tumors (tumors without all three molecular markers) receive chemotherapy alone [5]. Previous studies have indicated that patients with triple-negative breast cancer have worse clinical outcomes compared with hormone-receptor-positive (ER+ and PR+) and ERBB2-positive (ERBB2+) breast cancer patients [6,7,8]. Systemic chemotherapy for patients with triple-negative breast cancer causes acute and chronic toxicities, including nausea, vomiting, neuropathy, sterility, and congestive heart failure [9,10]. Therefore, there is still an urgent need to optimize the current therapeutic strategy for patients with triple-negative breast cancer.

Numerous natural dietary phytochemicals have been observed to inhibit carcinogenesis and have attracted growing interest as complementary and alternative medicines as well as for cancer chemoprevention due to their wide availability, mildness, and low toxicity [11]. *Glycyrrhiza* species (licorice) are widely used as herbal medicine in Asia, as they exhibit highly effective antitussive, expectorant, and antipyretic activities [12,13]. Among the bioactive ingredients isolated from licorice, isoliquiritigenin (ISL; 2′,4,4′-trihydroxychalcone) has been reported to exert considerable biological activities. ISL has an anti-inflammatory effect on the inhibition of nucleotide-binding domain leucine-rich repeat (NLR) and pyrin domain containing receptor 3 (NLRP3) inflammasome-associated inflammatory diseases [14,15]; an antioxidative effect on the activation of the Nrf2-induced antioxidant system [16]; a hepatoprotective effect against CCl4-induced liver injury [17]; and a cardioprotective effect on the reduction of oxidative stress [18].

ISL has also been reported to exert potential antitumor activity on multistage carcinogenesis procession in cervical [19], ovarian [20], prostate [21], and lung cancers [22]. Previously, we found that ISL of 10 μM exhibits an inhibitory effect on Vascular endothelial growth factor (VEGF)-induced triple-negative breast cancer migration and invasion through downregulation of the PI3K-AKT-MAPK signaling pathway [23]. We also observed that a dose of ISL over 25 μM showed an antiproliferation effect on breast cancer cells [23]. Hence, in the present study, we used an in vitro culture system to explore the molecular mechanism underlying ISL-induced antiproliferation of triple-negative MDA-MB-231 breast cancer cells. Moreover, an MDA-MB-231 tumor xenograft mouse model was generated to examine the preventive effect of ISL on tumor growth in vivo.

## 2. Materials and Methods

### 2.1. Cell Line and Culture Condition

Human breast cancer MDA-MB-231 cell line was purchased from the Bioresource Collection and Research Center (BCRC, Hsinchu, Taiwan). MDA-MB-231 cells were maintained in Dulbecco’s Modified Eagle Medium (DMEM)/F12 medium (Sigma-Aldrich, St. Louis, MO, USA) supplemented with 10% fetal bovine serum (FBS; CORNING, Manassas, VA, USA), 100 units/mL of penicillin, 100 μg/mL of streptomycin (CORNING), sodium bicarbonate (2.438 g/L; BioShop, Burlington, ON, Canada), and 4-(2-hydroxyethyl)piperazine-1-ethanesulfonic acid (HEPES; 5.986 g/L; BioShop) in a humidified incubator (37 °C, 5% CO_2_). Dimethyl sulfoxide (DMSO) was used as a solvent of ISL, stock concentration was 100 mM, and the dosage was according to a previous study [24].

### 2.2. MTT Assay

MDA-MB-231 cells were seeded in 96-well plates (2.5 × 10^3^ cells/well) and treated with ISL (Sigma-Aldrich, St. Louis, MO, USA) for 24, 48, and 72 h. The cell survival rate was analyzed using an MTT (3-(4,5-dimethyl-2-thiazolyl)-2,5-diphenyl-2H-tetrazolium bromide; Abcam, Cambridge, MA, USA) assay. At the end of incubation, serum-free DMEM medium with 0.5 mg/mL of MTT was substituted for conditional medium and incubated for an additional 3 h; subsequently, the media were removed. Crystal formazan was dissolved in 100 μL/well dimethyl sulfoxide (DMSO; ECHO Chemical Co. Ltd., Taipei, Taiwan). The optical density was measured by using a VERSA Max microplate reader (Molecular Devices, San Jose, CA, USA) at 570 and 630 nm as reference wavelengths.

### 2.3. Lactate Dehydrogenase (LDH) Assay

MDA-MB-231 cells were seeded in 96-well plates (2 × 10^4^ cells/well) and treated with ISL for 24, 48, and 72 h. The medium LDH activity was detected using the LDH Cytotoxicity Assay Kit (Cayman Chemical Company, Ann Arbor, MI, USA). The procedure was performed according to the manufacturer’s protocols. The absorbance was read at 450 nm with a VERSA Max microplate reader (Molecular Devices, San Jose, CA, USA).

### 2.4. Cell Counting

MDA-MB-231 cells (10^5^ cells) were seeded in 6 cm culture dishes. After attaching overnight, cells were treated with ISL for 48 h. Cell morphologies were photographed using a light microscope (Olympus, Tokyo, Japan). Then, cells were detected by trypsinization and resuspended in the culture medium. Cell suspensions were mixed with 0.4% trypan blue solution (Gibco, Grand Island, NY, USA). The number of cells was counted using a hemocytometer under an inverted phase-contrast microscope at 200× magnification.

### 2.5. Flow Cytometry Analysis of Cell Cycle Distribution and Apoptosis

To analyze the cell cycle distribution, MDA-MB-231 (1 × 10^5^) cells were seeded into 6 cm culture dishes. After attaching overnight, cells were treated with the vehicle or ISL at 25 and 50 μM in DMEM/F12 medium containing 10% FBS for 48 h. At the end of incubation, cells were detached by trypsinization. For the cell cycle distribution assay, the cells were fixed in 70% alcohol at −20 °C and stained with a propidium iodide (PI) solution (2 mg of DNAse-free RNAse A and 0.4 mL of 500 μg/mL PI was added to 10 mL of 0.1% Triton X-100 in phosphate buffered saline (PBS)) at room temperature for 30 min. For the apoptosis analysis, a commercial annexin V fluorescein isothiocyanate (FITC) apoptosis detection kit (BD Biosciences, San Jose, CA, USA) was used. The procedure was performed according to the manufacturer’s protocols. The cell cycle distribution and apoptosis were analyzed using a BD FACSCanto flow cytometer (BD Biosciences, San Jose, CA, USA). A minimum of 10,000 cells per sample were collected and further analyzed using CellQuest software (BD Biosciences).

### 2.6. Protein Preparation and Western Blot Analysis

Cells were lysed in RIPA lysis buffer containing protease and phosphatase inhibitors (Roche, Mannheim, Baden-Württemberg, Germany). Protein was quantitated by the Bradford assay and then resolved using sodium dodecyl sulfate polyacrylamide gel electrophoresis (SDS-PAGE). After transfer to a polyvinylidene Fluoride (PVDF) membrane, 1.5% Bovine serum albumin (BSA) solution was used to block the empty space of the membrane. Subsequently, membranes were incubated with primary antibodies—CDK4 (1:1000; Cell Signaling, Boston, MA, USA), cyclin D1 (1:1000; Cell Signaling), Bcl-2 (1:1000; Cell Signaling), Bax (1:1000; Cell Signaling), caspase-3 (1:1000; Cell Signaling), PARP (1:1000; Cell Signaling), GAPDH (1:10,000; Proteintech, Rosemont, IL, USA), p62 (1:1000; Cell Signaling), LC3 (1:1000; Cell Signaling), Beclin1 (1:10,000; Novus Biologicals, Littleton, CO, USA), Caspase-8 (1:1000; Cell Signaling), and Cathepsin B (1:1000; Cell Signaling) and β-actin (1:1000; Santa Cruz Biotechnology, Santa Cruz, CA, USA)—at 4 °C overnight and then with a horseradish peroxidase (HRP)-conjugated secondary antibody (1:10,000) for 1 to 2 h. The visual signal was captured by an image analysis system (UVP BioChemi, Analytik Jena US, Upland, CA, USA). The band densities were determined as arbitrary absorption units using the Image-J software program Version 1.52t (NIH, Bethesda, MD, USA). The expression level of these target proteins was analyzed by three individual experiments. In the same quantitative protein sample, two different molecular weights of the target protein were determined by the same gel. The membrane was cut into two fractions and incubated with two different antibodies at the same time.

### 2.7. Cell Transfection

The siRNA of SQSTM1/p62 (SignalSilence^®^ SQSTM1/p62 (Cell Signaling) were transfected into MDA-MB-231 cells with lipofectamine 3000 (Thermo Fisher Scientific, Waltham, MA, USA) reagent according to the manufacturer’s instructions. Briefly, 100 nM sip62 was transfected into MDA-MB-231 and treated with 50 μM ISL for 48 h.

### 2.8. Tumor Xenograft Mouse Model

The animal studies were conducted according to the protocols approved by the Institutional Animal Care and Use Committee (IACUC) of China Medical University (permit number: 99-190-C). In this study, 5-week-old female Nude-Foxn1^nu^ mice were purchased from BioLASCO (Taipei, Taiwan). The mice were housed with 12 h of artificial illumination in a temperature-controlled room (22 ± 2 °C). Food and water were provided ad libitum. Mice were randomly divided into two groups: (1) mice treated with PBS (0.25 mL/mouse) by oral gavage once daily for 2 weeks as a control, and (2) mice pretreated with ISL (Sigma-Aldrich) at 2.5 or 5.0 mg/mL by oral gavage (0.25 mL/mouse) once daily for 2 weeks. After pretreatment for 2 weeks, mice were inoculated with 100 μL PBS/mouse only as the control or human breast MDA-MB-231 cancer cells (5 × 10^6^ cells in 100 μL PBS/mouse) by a subcutaneous (s.c.) injection on the right flank, and they were constitutively treated with ISL for an additional 25 days. After implantation for 1 week, tumor volume was measured using calipers and the following formula was calculated: 0.5 × length × width^2^. At the end of the experiment, the mice were sacrificed by an intraperitoneal (i.p.) injection with an anesthetic mixture (30 μL/mouse; Zoletil:Rompun (*v*/*v*) = 1:1). The tumor tissues were weighed, photographed, and then fixed with 10% formalin for further immunohistochemistry (IHC) analysis.

### 2.9. IHC Staining

After deparaffinization, antigen retrieval, and blocking of peroxidase activity, the tissue sections were incubated with anti-Ki-67 antibody (1:200; Abcam, Cambridge, UK), anti-caspase-3 antibody (1:100; Cell Signaling), and anti-p62 antibody (1:100; Cell Signaling) at 4 °C overnight, separately. Slides were further incubated for 30 min with Super Enhance and polymer HRP. The bound antibody was elected by a species-specific secondary antibody and then developed with a 3-amino-9-ethylcarbazole (AEC) substrate. Images for IHC staining were captured with an EVOS^®^ microscope (Thermo Fisher Scientific, Waltham, MA, USA).

### 2.10. Statistical Analysis

All quantitative results are expressed as mean ± standard error of the mean (SEM), which were analyzed using Prism version 6.0 software (GraphPad, San Diego, CA, USA). The statistically significant difference from the respective controls for each experiment was determined using one-way analysis of variance (ANOVA) for all groups. Student’s unpaired *t*-test was used for comparison between two groups. Significance was accepted at * *p* < 0.05 and ** *p* < 0.01.

## 3. Results

### 3.1. ISL Suppressed Breast Cancer MDA-MB-231 Cell Growth

**Figure 1:**
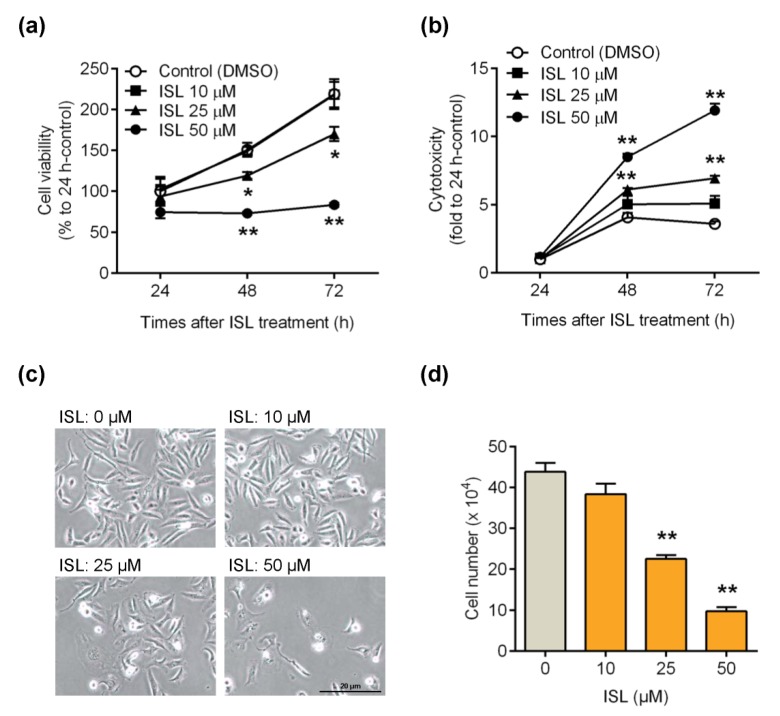
Inhibitory effects of isoliquiritigenin (ISL) on the proliferation of breast cancer MDA-MB-231 cells. (**a**) MDA-MB-231 cells were seeded in 96-well plates (3000 cells per well) with 100 μL per well culture medium. Cells were treated with ISL in various doses for 24, 48, and 72 h. At the end of incubation, cell viability was measured by MTT assay (*n* = 3). (**b**) MDA-MB-231 cells (2 × 10^4^ cells per well) seeded in 96-well plates. Cells were treated with various doses for 24, 48, and 72 h. The cytotoxic effects of ISL was detected using the lactate dehydrogenase (LDH) assay kit (*n* = 3). (**c**,**d**) MDA-MB-231 cells (2 × 10^5^ cells per well) were seeded in six-well plates. After treatment of cells with ISL for 48 h, the cell number was monitored (*n* = 4). Data are represented as mean ± SEM. * *p* < 0.05, ** *p* < 0.01 compared with the control group.

The MTT assay was performed to examine whether ISL can suppress breast cancer MDA-MB-231 cell growth. The results showed that cell viability was reduced by treatment of cells with ISL at 25 and 50 μM for 48 and 72 h (Figure 1a), whereas treatment of cells with ISL at 25 and 50 μM for 48 and 72 h was shown to increase cytotoxicity by LDH activity analysis (Figure 1b). The cell morphology and number were also monitored, and we found that the cell morphology switched from the spindle type to irregular by treatment of cells with ISL at 25 and 50 μM for 48 h (Figure 1c). Furthermore, a reduction of the cell number was observed by treatment of cells with 25 and 50 μM for 48 h (Figure 1d).

### 3.2. ISL Suppressed the Cell Cycle Progression of MDA-MB-231 Cells

**Figure 2:**
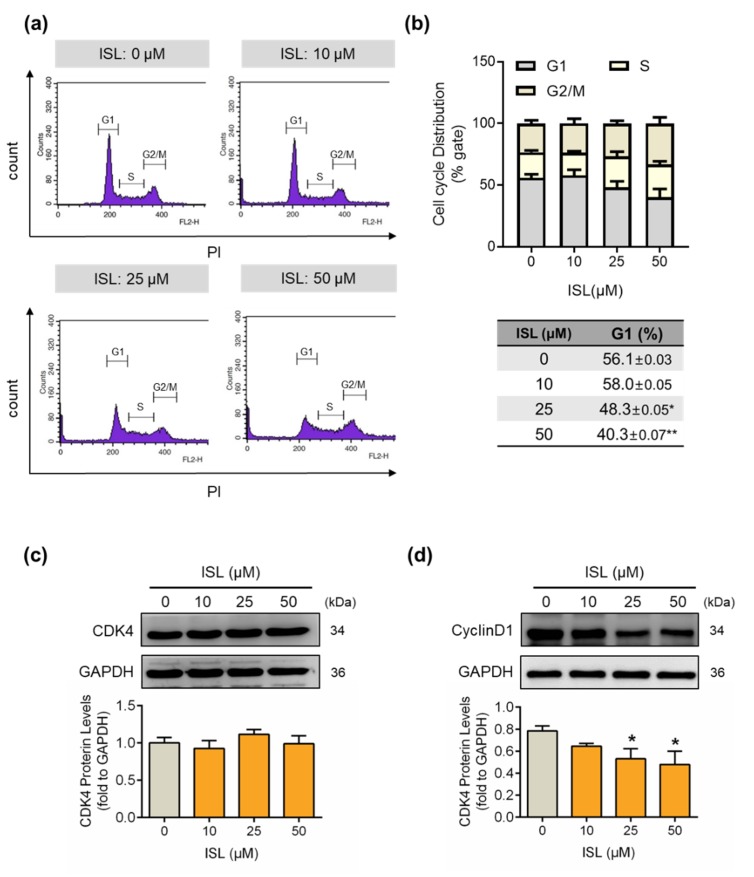
Cell cycle progression of MDA-MB-231 cells was suppressed by treatment with ISL. (**a**) MDA-MB-231 cells were treated with ISL for 48 h. Cells were stained with propidium iodide, and the cell cycle distributions were analyzed by the flow cytometry. (**b**) The quantitative results of cell distributions in MDA-MB-231 cells are shown. Cell-cycle-associated proteins (**c**) CDK4 and (**d**) CyclinD1 were analyzed using Western blot. Each target protein was normalized to GAPDH expression. Data are represented as mean ± SEM (*n* = 3). * *p* < 0.05, ** *p* < 0.01 compared with the control group.

Here, we studied whether ISL suppressed cell growth by inflecting the cell cycle progression. MDA-MB-231 cells were treated with ISL and then the cell cycle distribution was monitored using flow cytometry analysis. The results showed that treatment with ISL at 25 and 50 μM for 48 h reduced the cell population in the G1 phase in MDA-MB-231 cells (Figure 2a,b). In addition, the expression of G1/S gate-associated proteins CDK4 and cyclin D was detected. The results showed that treatment with ISL did not alter the expression of CDK4 (Figure 2c), but the expression of cyclin D1 was reduced by treatment with ISL at 25 and 50 μM for 48 h (Figure 2d).

### 3.3. ISL Induced Apoptotic Cell Death

**Figure 3:**
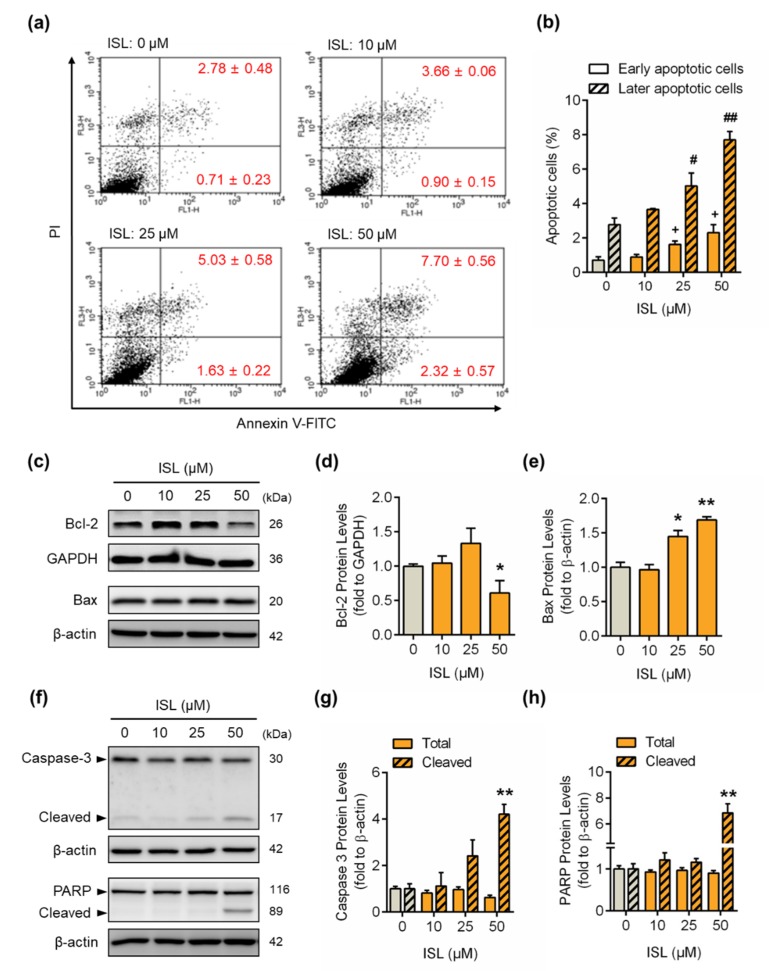
ISL induced cell apoptosis by upregulating apoptotic protein expression in MDA-MB-231 cells. (**a**) MDA-MB-231 cells were treated with ISL for 48 h. Cells were stained with propidium iodide and annexin V fluorescein isothiocyanate (FITC), and the apoptosis rates were analyzed by flow cytometry. (**b**) The quantitative data of apoptotic cell death in early and late phases are shown. (**c**) After treatment as indicated above, the anti- and proapoptotic proteins were monitored using Western blotting in MDA-MB-231 cells. (**d**,**e**) Each target protein was normalized to GAPDH or β-actin expression. (**f**) The expression of caspase-3 and its downstream molecule, PAPR, was monitored using Western blotting in MDA-MB-231 cells. (**g**,**h**) Each target protein was normalized to β-actin expression. Data are represented as mean ± SEM (*n* = 3). * *p* < 0.05, ** *p* < 0.01 compared with the control group. ^+^ *p* < 0.05 compared with the early apoptotic phase of the control group. ^#^ *p* < 0.05, ^##^ *p* < 0.01 compared with the later apoptotic phase of the control group.

To further examine whether ISL induced cell apoptosis in MDA-MB-231 cells, the cells were stained with annexin V FITC, and then the apoptotic cell population was analyzed by flow cytometry. The results showed that the annexin-V-positive (early apoptotic phase) and annexin-plus-PI-positive (later apoptotic phase) cell populations were increased by ISL treatment at 25 and 50 μM for 48 h (Figure 3a,b). The expression of Bcl-2 protein decreased from ISL treatment at 50 μM for 48 h (Figure 3c,d), whereas treatment with ISL increased the expression of Bax protein (Figure 3c,e). In addition, the cleaved form of caspase-3 was induced by treatment with ISL at 25 and 50 μM for 48 h (Figure 3f,g), and the expression of cleaved PARP was also increased by treatment with ISL at 50 μM for 48 h (Figure 3f,h).

### 3.4. ISL-Mediated p62 Accumulation Causes Autophagy-Mediated Apoptosis

**Figure 4:**
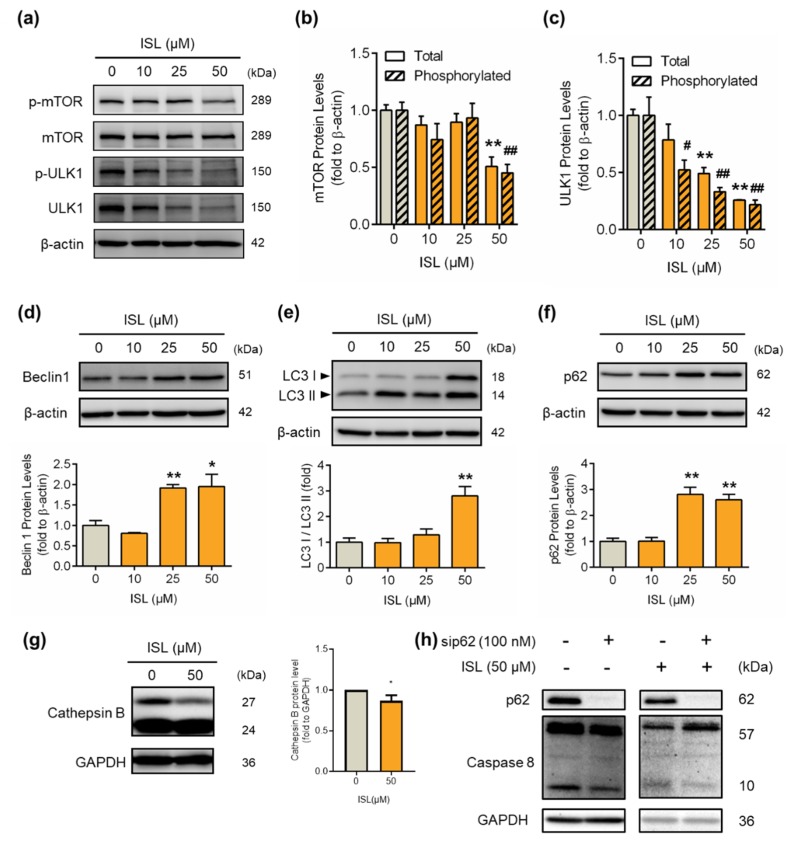
ISL treatment induced the expression of autophagy-associated proteins in MDA-MB-231 cells. MDA-MB-231 cells were treated with ISL for 48 h. (**a**) The expression levels of mTOR and ULK1 protein were analyzed by Western blotting. The total and phosphorylated forms of (**b**) mTOR and (**c**) ULK1 were normalized to β-actin expression. The expression levels of (**d**) p62, (**e**) Beclin1, (**f**) LC3, and (**g**) Cathepsin B were also analyzed using Western blotting. MDA-MB-231 cells were used lipofectamine 3000 (Thermo Fisher Scientific) to transfect SignalSilence^®^ SQSTM1/p62 (Cell Signaling), and treated with ISL for 48 h. The expression of (**h**) caspase-8 proteins were also analyzed using Western blotting. Data are represented as mean ± SEM (*n* = 3). * *p* < 0.05, ** *p* < 0.01 compared with the control group. ^#^ *p* < 0.05, ^##^ *p* < 0.01 compared with the phosphorylated protein level of the control group.

The total and phosphorylated protein levels of mTOR decreased after treatment with ISL at 50 μM for 48 h (Figure 4a,b). The downstream molecule ULK1 was activated by mTOR regulation in the autophagy pathway [25]. We found that the total and phosphorylated protein levels of ULK1 decreased after ISL treatment at 10, 25, and 50 μM for 48 h (Figure 4a,c). The autophagosome-associated proteins p62, Beclin1, and LC3 were increased by treatment with ISL for 48 h (Figure 4d–f). In our results observing that p62 accumulation, with autophagy inhibitor bafilomycin (BAF), blocked p62 accumulation and caused cell toxicity (Supplementary Figure S1). In the autophagy process, lysosomal proteases are needed, such as Cathepsins degrades p62 [26]. Treatment with ISL at 50 μM for 48 h significant decreased cathepsin B protein expression (Figure 4g), which explains the p62 accumulation. Moreover, p62 accumulation was related to the increased apoptosis through the increase of caspase-8. Furthermore, we used siRNA to eliminate p62 function. After 48 h, ISL increased caspase-8 protein expression. However, ISL combined with siRNA treatment have a lower expression of caspase-8 than ISL treatment (Figure 4h).

### 3.5. Preventive Effects of ISL on Breast Cancer Cell Growth in a Xenograft Mouse Model 

**Figure 5:**
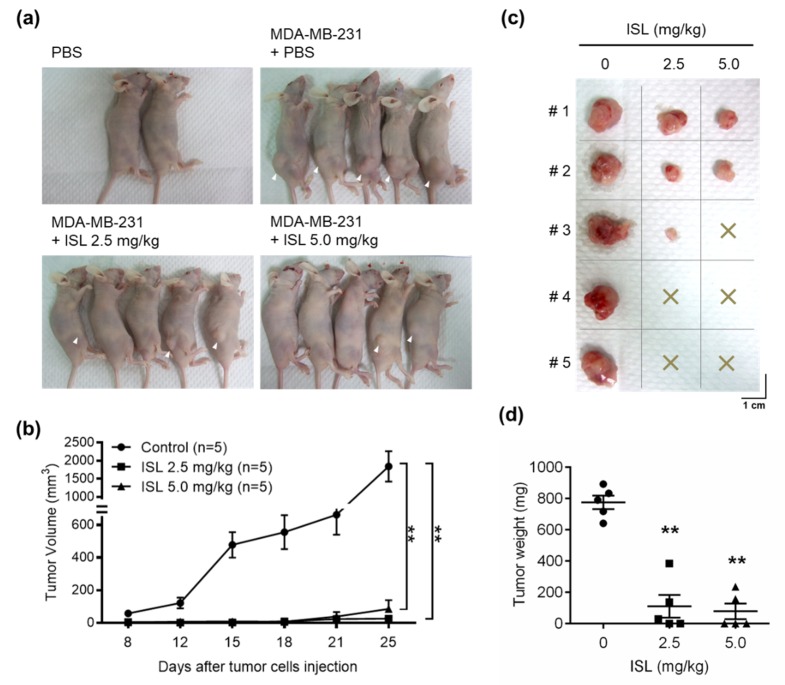
ISL suppressed tumor growth, as shown by the human MDA-MB-231 breast tumor xenograft mouse model. Mice were pretreated with ISL at 2.5 and 5.0 mg/kg or PBS as control by oral gavage once daily for 2 weeks. Then, MDA-MB-231 tumor cells (5 × 10^6^ cells per mouse) were implanted into mice by subcutaneous injection on the right flank and the mice received oral gavage of PBS and/or ISL for 25 days. At the end of the experiment, (**a**) the mice were photographed, (**b**) tumor size was monitored, (**c**) tumors were isolated and photographed, and (**d**) tumor weight was recorded. Data are represented as mean ± SEM (*n* = 5). ** *p* < 0.01 compared with the control group.

**Figure 6:**
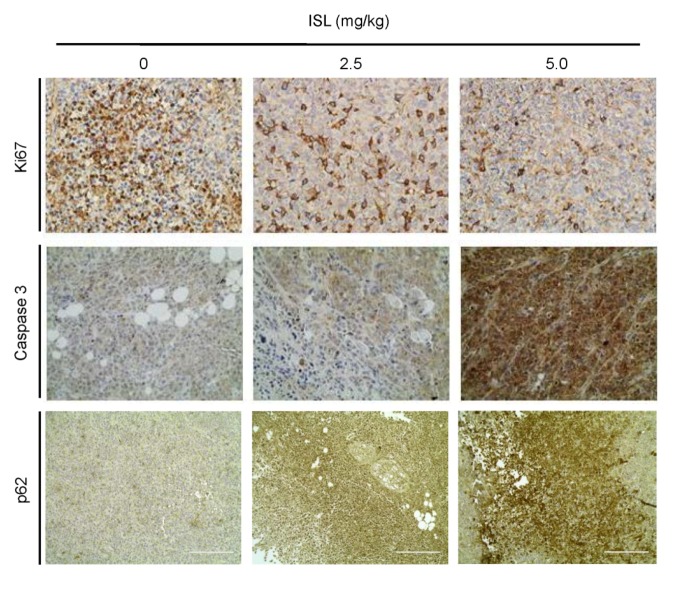
Representative results of IHC staining for Ki-67, caspase-3, and p62 protein expression levels in ISL-treated MDA-MB-231 breast tumor xenograft mouse model. MDA-MB-231 tumor cells (5 × 10^6^ cells per mouse) were implanted and mice were treated with ISL for 25 days Then, tumor tissues were isolated and immunohistochemistry was performed to analyze Ki-67, caspase-3, and p62 protein levels. Images were photographed (200× magnification; scale bar = 200 μm).

A breast cancer MDA-MB-231 cell xenograft mouse model was generated to evaluate whether ISL has a potential role in antitumor proliferation in vivo. After pretreatment with PBS or ISL at 2.5 and 5.0 mg/kg for 2 weeks, MDA-MB-231 cells were inoculated into mice, which were constitutively treated with ISL for an additional 25 days. The results showed that MDA-MB 231 tumors formed in all five mice by treatment with PBS. However, the number of MDA-MB 231 tumors was reduced by pretreatment with ISL at 2.5 mg/kg (three of five mice) and 5.0 mg/kg (two of five mice) (Figure 5a,c). Measuring the tumor volume elicited by MDAMB-231 cells showed that the tumor volume increased by treatment with PBS in a time-dependent manner, and ISL treatments significantly reduced them (Figure 5b). At the end of the experiment, tumor tissues were isolated and weighed. Treatments of mice with ISL at 2.5 and 5.0 mg/kg showed significantly reduced tumor weight compared with the vehicle group (Figure 5d). According to the IHC results, expression of the Ki-67 protein level was observed in tumor tissues for mice treated with ISL at 2.5 and 5.0 mg/kg, separately (Figure 6, top panel). Treatment with ISL at 5.0 mg/kg increased the caspase-3 expression level in tumor tissues (Figure 6, middle panel). Moreover, the expression of p62 was increased in tumor tissues by treatment with ISL at 2.5 and 5.0 mg/kg, separately (Figure 6, bottom panel).

### 3.6. ISL Suppressed VEGF Production and Capillary-Like Tube Formation of SVEC4-10 Cells

IHC analysis was performed to evaluate the expression of VEGF in tumor tissues by treatment with ISL. The results showed that the expression of VEGF was significantly reduced in treatment of mice with ISL at 2.5 and 5.0 mg/kg, separately (Supplementary Figure S2a). Measuring the serum VEGF level showed that treatment with ISL at 2.5 and 5.0 mg/kg reduced the serum VEGF level compared with the vehicle group (Supplementary Figure S2b). Additionally, to further investigate whether ISL has a potential role in antiangiogenesis, we performed a capillary-like tube formation assay. The results showed that the capability of capillary-like tube formation in SVEC4-10 cells was inhibited by treatment with ISL at 10 μM for 5 h (Supplementary Figure S3).

## 4. Discussion

Patients with triple-negative breast cancer are insensitive to endocrine therapy and ERBB2-targeted antibody treatment. Systemic chemotherapy for patients with triple-negative breast cancer is the predominant clinical treatment strategy [27,28]. Triple-negative breast cancer cells have been reported to exhibit genetic and phenotypical shifts in subpopulations, leading to resistance to the available standard drugs [29,30]. However, the functional differences made by genomically heterogeneous triple-negative breast cancer cells to chemoresistance remain poorly understood [31]. Natural ingredients from plants are widely explored to identify their availability for application in patients with breast cancer as an adjuvant approach [32]. For example, curcumin, a bioactive ingredient of the plant *Curcuma longa*, exerts an inhibitory effect on triple-negative MDA-MB-231 breast cancer cells through upregulation of p21 protein expression and enhancement of the Bax-to-Bcl-2 ratio [33,34]. A clinical phase I trial of curcumin has been reported to increase the sensitivity of breast cancer patients to docetaxel chemotherapy [35]. Similar effectiveness was observed for licorice roots (*Glycyrrhiza glabra*). The compounds derived from licorice have been widely investigated for their anticancer effects on several cancer cell types. Among the chalcone-type derivatives from licorice, ISL has been identified for cancer prevention in the last decade [12,36].

In the present study, we found that treatment of triple-negative MDA-MB-231 breast cancer cells with ISL inhibited cancer cell growth through blocking the cell cycle by reduction of cyclin D1 expression. The enhancement of the sub-G1 cell population by ISL treatment indicated that the apoptotic cell death pathway was active. Flow cytometry analysis was used to further confirm whether ISL induced MDA-MB-231 cell apoptosis. Our results showed that treatment with ISL increased the annexin-V- and PI-positive cell population. The expression of the anti-apoptotic protein Bcl-2 was reduced, whereas expression of the proapoptotic protein Bax was increased. Furthermore, the downstream molecules caspase-3 and PARP were active. In agreement with our findings, Peng et al. reported that treatment of MDA-MB-231 cells with ISL induced cell apoptosis, and they observed an enhancement of the ratio of Bax to Bcl-2 by ISL treatment [37]. Moreover, ISL treatment was also observed to induce cancer cell apoptosis in lung cancer [38], uterine leiomyoma [39], and endometrial cancer [40].

A clinical study reported that the expression of Beclin1 is lower in patients with breast and ovarian cancers [41]. Beclin1, a Bcl-2 homology 3 (BH3)-domain-only protein, is an important protein in initial autophagy. The anti-apoptotic Bcl-2 protein has been reported to interact with Beclin1 via its BH3 domain [42]. The Beclin1 and Bcl-2 complex suppresses the pre-autophagosomal structure combination, thereby inhibiting autophagy progression. In this study, the results showed that treatment with ISL reduced the expression of anti-apoptotic Bcl-2 and induced autophagy-induced- apoptosis. Our results found that ISL may have p62 accumulation. Additionally, p62 may activate caspase-8, which can induce apoptosis by autophagy inhibition. Caspase-8 activation promotes cell apoptosis [43]. In addition, we found that treatment with ISL induced autophagy-associated signaling proteins mTOR and ULK1 were downregulated by ISL treatment, while the expressions of p62, Beclin1, and LC3 autophagy-promoting proteins were all increased. However, during autophagy progression, p62 was enclosed by autolysosome and degraded by cathepsin B in lysosomes [44], although both p62 and LC3 II levels were increased by ISL. Therefore, ISL influences p62 accumulation by reduced cathepsin B to inhibit autophagy by blocked phagolysosome formation. Furthermore, ISL can activate apoptosis by increased caspase-8 protein expression.

Cathepsin B is a lysosomal protease and is related to autophagy progression and p62 degradation [45]. Cancer studies have shown that cathepsin B is related to cancer cell proliferation [46], and its ability to break down the cell base membrane can increase cancer cell invasion. Our results show that ISL can decrease cathepsin B protein expression to suppress p62 accumulation, increasing autophagy-mediated apoptosis and decreasing cancer cell proliferation-related Ki67 expression.

Cuendet et al. reported that administration of ISL for 1 week delays 7,12-dimethylbenz(*a*)anthracene (DMBA)-induced mammary carcinogenesis in female rats. However, this effect does not reduce the incidence of tumor formation [36,47]. In the present study, to examine whether ISL has preventive effectiveness for anti-proliferation of breast cancer cells, mice were administered with ISL by oral gavage for 2 weeks before implantation of MDA-MB-231 breast cancer cells. Our results showed that pretreatment of mice with ISL reduced the success rate of breast cancer cell implantation, thereby reducing tumor tissue formation. Despite tumor formation from the ISL-treated group, the expression of the Ki-67 protein, a hallmark of proliferation, was reduced, whereas the expressions of caspase-3 and p62 proteins were increased, suggesting that these tumors were undergoing apoptotic and autophagic cell death progression. In addition, we further evaluated the effects of ISL on anti-angiogenesis in breast cancer cells. Our results show that the protein expression and secretion of VEGF were reduced by treatment of mice with ISL. Consistent with our results, Wang et al. reported that treatment with ISL suppresses VEGF-induced proliferation of human umbilical vein endothelial cells (HUVECs) through interaction with the VEGF2R receptor and the reduction of VEGF-regulated downstream signaling [48]. Moreover, we also found that the capability of capillary-like tube formation of SVEC4-10 cells was abolished by treatment with ISL. ISL has a potential role in antiangiogenesis, which might contribute to the inhibitory effect of ISL on the migration and invasion ability of cancer cells.

The effectiveness of ISL in combination with traditional chemotherapeutic drugs has also been investigated. Wang et al. reported that ISL exerts synergistic effects with first-line chemotherapeutic drugs for breast cancer therapy [49]. Specifically, ISL chemosensitizes breast cancer stem cells by docking into the adenosine triphosphate (ATP) domain of GRP78 directly, which in turn suppresses β-catenin/ATP-binding cassette subfamily G2 (ABCG2) signaling [49]. The side-effects of systemic chemotherapeutic therapy were also investigated. ISL has been reported to enhance the toxicity-induced cell death of bladder cancer cells. Furthermore, ISL has been shown to attenuate cisplatin-induced normal renal proximal tubular cell death via activation of the nuclear factor (erythroid-derived 2)-like 2 (Nrf2)/heme oxygenase-1 (HO-1) regulatory pathway [50]. Similarly, the protective effect of ISL on the kidney and liver against cisplatin-induced colon cancer cell death has also been demonstrated [51]. These results suggest that the promotion of chemosensitivity and the lower toxicity of ISL should be further considered in clinical trials.

## 5. Conclusions

**Figure 7:**
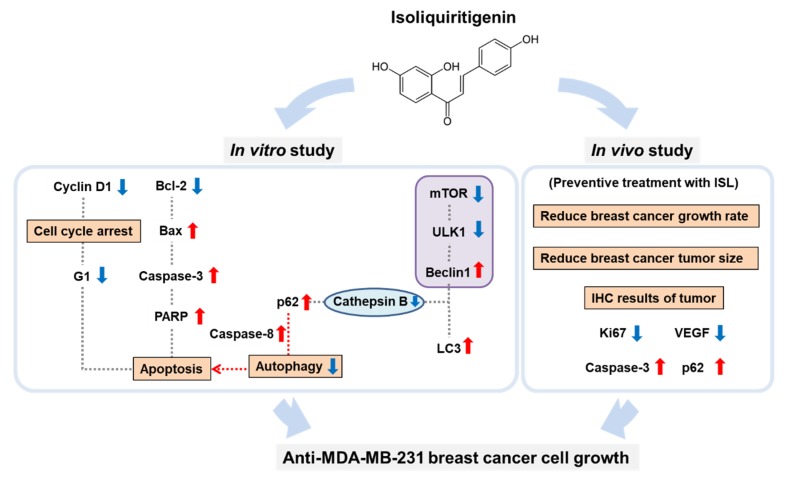
Schematic representation of the inhibitory effect of ISL on the proliferation of triple-negative breast cancer MDA-MB-231 cells through p62 accumulation and activation of apoptotic cell death programs. Blue arrow: decreased; Red arrow: increased.

In conclusion, the results of this study showed that ISL effectively inhibited triple-negative MDA-MB-231 breast cancer cells through the activation of apoptotic cell death and cause p62 accumulation activated autophagy mediated apoptosis cell death progressions. In the mouse breast cancer tumor xenograft model, the preventive effect of ISL on antitumor growth was demonstrated (Figure 7). Therefore, supplementation with ISL may be an effective anticancer approach for use in clinical trials.

## Supplementary Materials

The following are available online at https://www.mdpi.com/2076-3921/9/3/228/s1, **Figure S1**: ISL and bafilomycin A1 (BAF) treatment induced the expression of autophagy-associated proteins and caused cell toxicity. MDA-MB-231 cells were seeded in 96-well plates (3000 cells per well). Cell were pretreated with BAF for 3 h, and combined with ISL for 48 h. At the end of incubation, (**a**) cell viability was measured by MTT assay. MDA-MB-231 cells were pretreated with BAF for 3 h, and combined with ISL for 48 h. The expression of (**b**) p62 and (**c**) Beclin1 protein were analyzed using western blotting. Data were represented as means ± SD (*n* = 3). a, *p* < 0.05, compared with control group. b, *p* < 0.05, compared with ISL group. **Figure S2**: Effects of ISL on the expression and secretion of VEGF. MDA-MB-231 tumor cells (5 × 106 cells per mouse) were implanted and mice were treated with ISL for 25 days. At the end of experiment, (**a**) tumor tissues were isolated and performed immunohistochemistry to analyze VEGF protein level. Images were photographed at 200× magnification. (**b**) Serum VEGF levels were measured using ELISA. Data represent as means ± SEM (*n* = 5 each group). ** *p* < 0.01 compared with the control group. **Figure S3**: Effects of ISL on the capillary-like tube formation. ISL inhibited capillary-like tube formation of mouse endothelial cells SVEC4-10 cells in matrigel. Images were photographed at 100× magnification.
