# Supplementary material for: Dietary Compound Isoliquiritigenin, an Antioxidant from Licorice, Suppresses Triple-Negative Breast Tumor Growth via Apoptotic Death Program Activation in Cell and Xenograft Animal Models
Source: Antioxidants (Basel). 2020 Mar 10;9(3):228. doi: 10.3390/antiox9030228 (PMC7139602; doi:10.3390/antiox9030228)
Supplement: Supplementary file 1 [file antioxidants-09-00228-s001.pdf]

Dietary Compound Isoliquiritigenin, an Antioxidant from Licorice, Suppresses Triple-Negative Breast Tumor Growth via Apoptotic Death Program Activation in Cell and Xenograft Animal Models

Po-Han Lin [1], Yi-Fen Chiang [1,#], Tzong-Ming Shieh [2,,3#], Hsin-Yuan Chen [1], Chun-Kuang Shih [1], Tong-Hong Wang [4,5], Kai-Lee Wang [6], Tsui-Chin Huang [7], Yong-Han Hong [8], Sing-Chung Li [1] and Shih-Min Hsia [1,9,10,11*]

1 School of Nutrition and Health Sciences, College of Nutrition, Taipei Medical University, Taipei 11031, Taiwan; phlin@tmu.edu.tw (P.-H.L.); yvonne840828@gmail.com (Y.-F.C.); hsin246@gmail.com (H.-Y.C.); ckshih@tmu.edu.tw (C.-K.S.); sinchung@tmu.edu.tw (S.-C.L.)
2 School of Dentistry, College of Dentistry, China Medical University, Taichung 40402, Taiwan; tmshieh@mail.cmu.edu.tw
3 Department of Dental Hygiene, College of Health Care, China Medical University, Taichung 40402, Taiwan
4 Tissue Bank, Chang Gung Memorial Hospital, Tao-Yuan 33305, Taiwan; cellww@adm.cgmh.org.tw
5 Graduate Institute of Health Industry Technology, Chang Gung University of Science and Technology, Tao-Yuan 33305, Taiwan.
6 Department of Nursing, Ching Kuo Institute of Management and Health, Keelung City 20301, Taiwan; kellywang@tmu.edu.tw
7 Graduate Institute of Cancer Biology and Drug Discovery, College of Medical Science and Technology, Taipei Medical University, Taipei 11031, Taiwan; tsuichin@tmu.edu.tw
8 Department of Nutrition, I-Shou University, Kaohsiung City 82445, Taiwan; yonghan@isu.edu.tw
9 Graduate Institute of Metabolism and Obesity Sciences, College of Nutrition, Taipei Medical University, Taipei 11031, Taiwan
10 School of Food Safety, College of Nutrition, Taipei Medical University, Taipei 11031, Taiwan
11 Nutrition Research Center, Taipei Medical University Hospital, Taipei 11031, Taiwan.
* Correspondence: bryanhsia@tmu.edu.tw; Tel.: +886-2-2736-1661 (ext. 6558)
# These authors equal contribution as the first author in this study.

Supplemental Materials and Methods

Immunohistochemistry staining

After deparaffinization, antigen retrieval, and blocking of peroxidase activity, the tumor tissue sections were incubated with anti-VEGF antibody (1:200; Abcam, Cambridge, United Kingdom) at 4°C overnight. Slides were further incubated for 30 minutes with Super Enhance and polymer horseradish peroxidase (HRP). The bound antibody was elected by a species-specific secondary antibody, and then developed with 3-Amino-9-ethylcarbazole (AEC) substrate. Images for IHC staining were captured with The EVOS® microscope (Thermo Fisher Scientific).

Enzyme-linked immunosorbent assay (ELISA) of VEGF

At the end of experiment, mice were euthanized and bloods were collected from the orbital venous plexus. Serum was separated by centrifugation and the VEGF concentration was analyzed by using the Human Quantikine VEGF ELISA Kit (R&D Systems, Inc.; Minneapolis, MN, USA). All procedures were performed according to the manufacturer's protocols. Inter- and intra-assay coefficients of variance given by the manufacturer for cell culture supernatant assays are 6.2–8.8 and 4.5–6.7 %, respectively.

Tube formation assay

Mouse endothelial cells SVEC4-10 cell line was purchased from Bioresource Collection and Research Center (BCRC: #60220; Hsinchu, Taiwan). SVEC4-10 cells were incubated according to a protocol modified from one used previously [1]. Briefly, conditional media were collected from the equivalent cells

of MDA-MB-231 (4x10[5] cells) cultivated at 37°C for 24 hr. First, Matrigel (BD Biosciences; 354234) 50 µL/well was covered in 96-well plates and incubated at 37°C for approximately 1 hour for gel formation. SVEC4-10 cells (4x10[5]) were resuspended in 100 µl fresh culture medium on the colloid Matrigel. Then, conditional media form MDA-MB-231 cells were diluted (volume ratio=1:1) with fresh media and added into wells for SVEC4-10 culture. Random photographs were taken of each well from 0 to 5 hr.

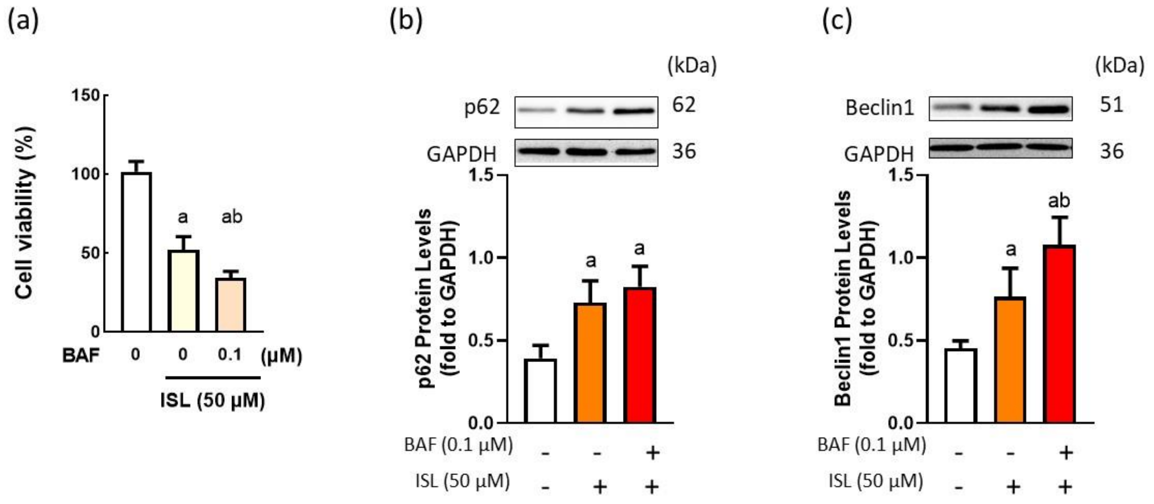

Supplementary Figure S1. ISL and bafilomycin A1 (BAF) treatment induced the expression of autophagy-associated proteins and caused cell toxicity. MDA-MB-231 cells were seeded in 96-well plates (3000 cells per well). Cell were pretreated with BAF for 3h, and combined with ISL for 48h. At the end of incubation, cell viability was measured by MTT assay. MDA-MB-231 cells were pretreated with BAF for 3h, and combined with ISL for 48h. The expression of (b) p62 and (c) Beclin1 protein were analyzed using western blotting. Data were represented as means ± SD. a, $p<0.05$, compared with control group. b, $p<0.05$, compared with ISL group.

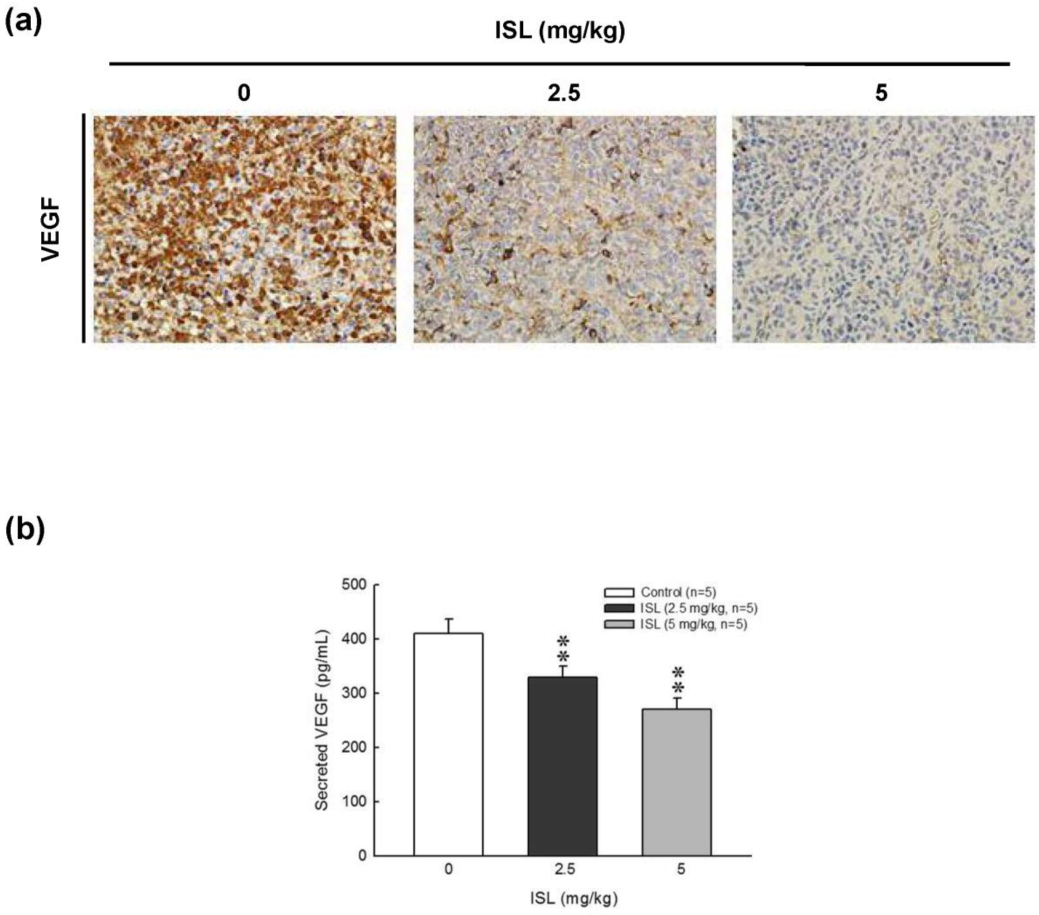

Supplementary Figure S2. Effects of ISL on the expression and secretion of VEGF. MDA-MB-231 tumor cells (5 x 10^6 cells per mouse) were implanted and mice were treated with ISL for 25 days. At the end of experiment, **(A)** tumor tissues were isolated and performed immunohistochemistry to analyze VEGF protein level. Images were photographed at 200x magnification. **(B)** Serum VEGF levels were measured using ELISA. Data represent as means ± SEM (n = 5 each group). ***P* < 0.01 compared with the control group.

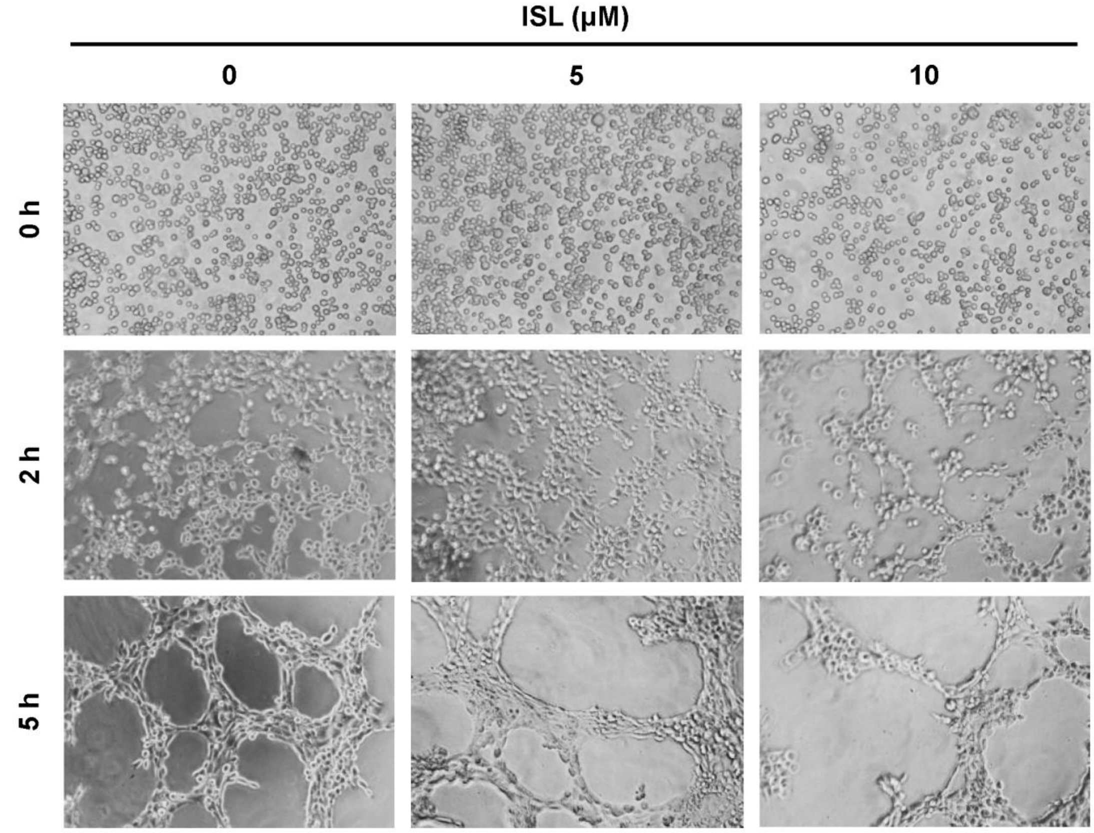

Supplementary Figure S3. Effects of ISL on the capillary-like tube formation. ISL inhibited capillary-like tube formation of mouse endothelial cells SVEC4-10 cells in matrigel. Images were photographed at 100x magnification.

References:

1. Shih, Y.H.; Chang, K.W.; Chen, M.Y.; Yu, C.C.; Lin, D.J.; Hsia, S.M.; Huang, H.L.; Shieh, T.M. Lysyl oxidase and enhancement of cell proliferation and angiogenesis in oral squamous cell carcinoma. *Head Neck* **2013**, *35*, 250-256, doi:10.1002/hed.22959.
